# Reflection at Night: Exploring University Students’ Cognitions Regarding Nighttime Destination Authenticity

**DOI:** 10.3390/bs16071094

**Published:** 2026-07-02

**Authors:** Zhilun (Alan) Huang, Songxue Zhang, Chunfeng Li, Kang-Lin Peng, Yuan Ye

**Affiliations:** 1 Faculty of International Tourism and Management, City University of Macau, Macau 999078, China; klpeng@cityu.edu.mo (K.-L.P.); t25091110277@cityu.edu.mo (Y.Y.); 2 School of Business, Nanfang College, Guangzhou 510970, China; 3 Faculty of Innovation and Design, City University of Macau, Macau 999078, China; u24092110383@cityu.edu.mo

**Keywords:** nighttime, destination authenticity, critical reflection, nocturnal escapism, psychological empowerment theory, cognition–affect–conation framework

## Abstract

Nighttime destinations, characterized by distinctive lighting, atmosphere, and activities, provide rich cognitive stimuli for university students. However, university students’ cognition regarding authenticity within such settings remains underexplored. Grounded in psychological empowerment theory, and the cognition–affect–conation framework, this study investigates how university students’ perceptions of objective and existential authenticity (i.e., intrapersonal and interpersonal) in a nighttime destination coincide with the meaning of nighttime destination and subsequent critical reflection. It further investigates the moderating role of nocturnal escapism between the meaning of nighttime destination and critical reflection. Using survey data from 764 university students at the “City of Sleepless in the Song Dynasty,” this research employs Partial Least Squares Structural Equation Modeling (PLS-SEM) and fuzzy-set Qualitative Comparative Analysis (fsQCA). The results indicate that intrapersonal authenticity shows the strongest association with the meaning of nighttime destination and critical reflection. The fsQCA reveals four distinct configurations consistently associated with high critical reflection, highlighting configurational complexity. This study offers insights into university students’ cognition of nighttime destination authenticity and discusses perceived experiential qualities that may coincide with critical reflection.

## 1. Introduction

With the rapid development of the nighttime economy, destinations characterized by cultural immersion and social interaction are increasingly integrated into the daily routines of university students (Li et al., 2025; Yin & Ni, 2025). Beyond entertainment, these settings provide rich sensory and cultural stimuli. While previous research has explored cognitive engagement in traditional settings such as museums (Chng & Tan, 2024; Ko, 2026; Uysal, 2025), empirical investigations into how students perceive the cognitive relevance of nighttime destinations remain limited. Specifically, there is a paucity of research examining how university students’ perceptions of destination authenticity in these nighttime contexts associate with their subjective experiences of critical reflection. Critical reflection is conceptualized as a higher-order cognitive activity involving the examination and reconstruction of existing assumptions (Kember et al., 2000). To address this gap, this study investigates the following research question: How do university students’ perceptions of nighttime destination authenticity coincide with their critical reflection through the association mediated by the meaning of nighttime destination, and how does nocturnal escapism moderate this pathway?

Within nighttime destinations, authenticity perception serves as a crucial cognitive construct underpinning students’ critical reflection. Drawing on the work of Kolar and Zabkar (2010) and Fu (2019), this study deconstructs destination authenticity into two layers: objective authenticity and existential authenticity. Objective authenticity refers to the historical accuracy and genuineness presented by the destination’s environment, architecture, and displays (T. Zhang & Yin, 2020). Existential authenticity, meanwhile, encompasses intrapersonal authenticity, which relates to the self-liberation, self-exploration, and self-realization individuals feel during the experience, and interpersonal authenticity, which involves the genuine and natural connections established with others (Yi et al., 2017). Although these dimensions have been identified, university students’ cognition of these different dimensions of authenticity, and the ways it relates to deep critical reflection, remain underexplored.

Building on this foundation, the present study introduces psychological empowerment theory to construct the core mediating mechanism. This theory suggests that the core of empowerment lies in individuals ascribing the meaning of nighttime destination to their environment and their own actions, and in perceiving a sense of control and influence over that environment (Llorente-Alonso et al., 2024). In the context of a nighttime destination, this experience of empowerment is reflected in the meaning of nighttime destination. When students perceive the authenticity of a nighttime destination, this perception can enhance their sense of engagement and identification with the experience, thereby helping them construct personalized meaning from it. The meaning of nighttime destination is not merely an emotional satisfaction but also a cognitive correlate, coinciding with critical reflection. For instance, research by Kang et al. (2017) indicates that an individual’s perception of meaning in an activity aligns with subsequent deep cognition and behavioral change. Therefore, the meaning of nighttime destination derived from authenticity perceptions may serve as a crucial affective factor in the association with university students’ critical reflection.

To systematically integrate the complete chain from cognition to final behavior, this study further adopts the cognition–affect–conation framework (Bagozzi, 1992). This framework characterizes the progression from cognitive appraisal of external stimuli to internal affective responses, and then to conative tendencies. Within the proposed model, this framework clarifies the cognitive profile of university students. It begins with the cognitive appraisal of nighttime destination authenticity. This appraisal coincides with a corresponding affective response, namely the formation of a positive perception of the meaning of nighttime destination. Ultimately, this positive meaning of nighttime destination is significantly associated with the critical reflection. Therefore, this framework provides a coherent theoretical logic for the model, connecting authenticity cognition to meaning affect and then to critical reflection conation. It is noteworthy that the effect of this cognitive–affective framework may vary across individuals. Consequently, this study introduces nocturnal escapism as a moderating variable. Nocturnal escapism is defined as an individual’s tendency to temporarily escape daily routines and pressures through nighttime activities and environments (Semrad & Rivera, 2018). For university students who often face significant academic and social pressures, nocturnal escapism represents an important psychological motive. This escapist tendency may strengthen the association between the meaning of nighttime destination and critical reflection.

Ultimately, building on psychological empowerment theory, and the cognition–affect–conation framework, this study developed a conceptual model and employed a two-phase quantitative design. A questionnaire survey was administered to university students visiting the “City of Sleepless in the Song Dynasty” in Taian, China, and the research hypotheses were tested using Partial Least Squares Structural Equation Modeling (PLS-SEM). Subsequently, to uncover the multiple antecedent configurations consistently associated with a high level of critical reflection, this study further applied fuzzy-set Qualitative Comparative Analysis (fsQCA). This study aims to examine the associations between multidimensional authenticity perceptions and critical reflection, specifically accounting for the mediating role of the meaning of nighttime destination and the moderating role of nocturnal escapism. Additionally, it aims to identify the configurations associated with heightened critical reflection among university students, offering a holistic perspective on the interplay of these factors. The theoretical contribution of this study lies in its integration of multiple theoretical perspectives, thereby deepening the understanding of university students’ cognitions within nighttime destinations. Its practical contribution is to provide theoretical guidance for designing nighttime experiences that integrate emotional appeal.

## 2. Literature Review

### 2.1. The Authenticity of Nighttime Destination

Destination authenticity is typically deconstructed into two core dimensions: objective authenticity and existential authenticity (Fu, 2019; Kolar & Zabkar, 2010). This framework provides crucial support for understanding the cognitive value of authenticity in nighttime destinations. Drawing on Wang (2007), objective authenticity refers to tourists’ perceptions of the historical accuracy and originality signaled by the material features of a leisure space. Although objective genuineness is a physical attribute, this study focuses on how visitors perceive the credibility of the architecture, objects, and environmental atmosphere (T. Zhang & Yin, 2020). In the context of nighttime destinations, this perception materializes through meticulously restored historical street scenes, period-specific lighting designs, and performances rooted in cultural narratives. These material cues evoke sensory and cognitive responses; as Kolar and Zabkar (2010) note, the perceived material attributes of a site are strongly associated with visitors’ cognitive engagement. For university students, who are at a critical stage of cognitive development, the perceived environmental authenticity of nighttime destinations serves as more than mere entertainment scenery. Perceiving the environment as authentic provides external validation for students. This perception is associated with moving beyond superficial consumption to scrutinize the alignment between the site’s cultural information and their prior knowledge, thereby encouraging critical reflection.

In contrast to objective authenticity, which focuses on the attributes of the object, existential authenticity shifts attention to the subjective psychological state and social interactions of the experiencer, emphasizing the sense of authentic being attained by the individual during the experience (Yi et al., 2017). This dimension can be further divided into intrapersonal authenticity and interpersonal authenticity. Intrapersonal authenticity refers to the feeling of self-liberation, authentic self-expression, and potential fulfillment that an individual experiences in leisure or tourism contexts (J. Zhang & Yang, 2025), such as breaking free from daily role constraints during free exploration in a nighttime destination to engage in self-dialog and discovery. Interpersonal authenticity is reflected in the genuine, natural, and deep connections experienced through interactions with others (Kernis & Goldman, 2005). Research by Fu (2019) confirms that experiences of existential authenticity coincide with an individual’s destination identification. Existential authenticity is associated with critical reflection among university students. On one hand, intrapersonal authenticity associates with lower psychological defensiveness, which correlates with students’ willingness to engage in open, even self-challenging, critical reflection regarding the information encountered through objective authenticity. On the other hand, deep social interaction linked to interpersonal authenticity, such as discussing a historical scene or cultural phenomenon with peers, represents a socially negotiated dynamic in the construction of the meaning of nighttime destination. This dynamic coincides with critical reflection through the collision of viewpoints and the negotiation of the meaning of nighttime destination.

### 2.2. Psychological Empowerment and Critical Reflection

Psychological empowerment theory shifts away from the traditional view of empowerment as an external grant of power or resources, emphasizing instead that empowerment is an internal psychological state (Zimmerman, 2000). The theory posits that genuine empowerment occurs when individuals feel they can exert influence over their environment and actions and perceive the activities in which they engage as holding personal value and meaning of nighttime destination (Llorente-Alonso et al., 2024). Its core lies in an individual’s confidence in their own abilities, a sense of control over their environment, and a clear understanding of the purpose of their activities (Oliveira et al., 2023). The individual’s perception of the meaning of nighttime destination serves as a crucial mediator. It is by constructing personalized meaning of nighttime destination from their experiences that individuals connect external activities to their internal value systems, which is associated with deeper psychological authorization and motivation, correlating with subsequent profound cognition.

Applying this theory to the context of nighttime destinations allows for an understanding of the specific pathway through which university students gain psychological empowerment. When students perceive authenticity within a nighttime destination, this perception associates with strengthened identification with and engagement in the environment and experience, which in turn relates to their construction of the meaning of nighttime destination from their visit. This perception of the meaning of nighttime destination is itself an outcome of psychological empowerment, transforming individuals from passive information recipients into active meaning-makers (Zimmerman, 2000). Moreover, this sense of conferred meaning of nighttime destination coincides with critical reflection. Consistent with Kang et al. (2017), the meaning of nighttime destination associates with critical reflection. Within a nighttime destination, when students find the experience personally meaningful, they are more likely to engage in thorough examination, questioning, and reconstruction of the cultural information, social interactions, and self-experience it contains, which constitutes the subjective experience of critical reflection.

### 2.3. Cognition–Affect–Conation Framework

The cognition–affect–conation framework is a classic psychological model that systematically delineates the sequential mental architecture through which an individual responds to an external stimulus and forms a behavioral tendency (Bagozzi, 1992). The framework begins with the cognitive appraisal of a stimulus, leads to an internal affective response, and culminates in a specific conative or behavioral intention. It provides a clear sequential logic for understanding the psychological mechanisms underlying complex behaviors. In the context of this study, which explores how nighttime destination authenticity relates to critical reflection, this framework offers a suitable theoretical pathway for integration. Specifically, it conceptualizes the psychological state of university students as an ordered chain: it starts with the cognitive appraisal of destination authenticity. This appraisal then evokes an individual-level affective outcome, namely the affective response towards the meaning of nighttime destination derived from the experience. Ultimately, this positive perception of the meaning of nighttime destination is significantly associated with the intention and actual action to engage in critical reflection. Therefore, the cognition–affect–conation framework provides the fundamental logical structure for building a complete theoretical model that connects authenticity cognition to the meaning of nighttime destination as affect, and then to critical reflection conation.

### 2.4. Hypothesis Development and Research Model

The perception of authenticity serves as a key cognitive starting point for individuals to engage in deep experiences and the construction of the meaning of nighttime destination. Specifically, objective authenticity, through its historically accurate material cues and narrative environment, provides university students with a credible and detailed cognitive framework. This enhances the perceived credibility and engagement in the experience, forming an important foundation for individuals to derive personal meaning of nighttime destination from it (Kolar & Zabkar, 2010; T. Zhang & Yin, 2020). In contrast, existential authenticity facilitates the generation of the meaning of nighttime destination from both intrinsic psychological and social dimensions. On the one hand, intrapersonal authenticity allows individuals to break free from daily role constraints during the experience, engaging in self-exploration and expression. This dynamic of self-dialog and liberation constitutes a source of deep meaning of nighttime destination (Fu, 2019). On the other hand, interpersonal authenticity, characterized by sincere and natural social interaction, creates a space for jointly negotiating and reinforcing the meaning of nighttime destination through the exchange of perspectives and emotional resonance (Yi et al., 2017). Therefore, the three dimensions of authenticity collectively provide complementary pathways that coincide with university students’ construction of personalized meaning of nighttime destination.

Furthermore, the authentic features of a nighttime destination act as a potent cognitive stimulus. For instance, the tension between objectively authentic historical presentations and personal experience, or an individual’s active pursuit of intrapersonal and interpersonal authenticity, can directly elicit cognitive appraisal, comparison, and questioning among university students. This direct cognitive engagement may prompt individuals to critically examine their own pre-existing understandings or social norms, potentially bypassing a more complex pathway of personal meaning internalization. Additionally, psychological empowerment theory posits that a sense of control and perceived influence over the environment is central to empowerment (Llorente-Alonso et al., 2024). When university students perceive the authenticity of an environment, this perception may itself grant them a sense of cognitive grounding and legitimacy for exploration, thereby directly motivating them to engage in deeper, more critical reflection. Therefore, this study puts forward the following hypotheses:

**H1.**  *The perceived [a] objective authenticity, [b] intrapersonal authenticity, and [c] interpersonal authenticity of nighttime destinations are positively associated with the meaning of nighttime destinations.*

**H2.**  *The perceived [a] objective authenticity, [b] intrapersonal authenticity, and [c] interpersonal authenticity of nighttime destinations are positively associated with critical reflection.*

The meaning of nighttime destination that individuals construct from their experiences serves as a key internal factor coinciding with psychological empowerment, which in turn aligns with deeper cognitive engagement and behavioral change (Llorente-Alonso et al., 2024). University students’ perception of the meaning of nighttime destination signals a shift from being passive recipients of information to active meaning-makers. This transformation itself is a core outcome of psychological empowerment. More importantly, this internalized meaning of nighttime destination is significantly associated with individuals engaging in critical reflection. This is supported theoretically by the cognition–affect–conation framework, which positions the meaning of nighttime destination as the key affective link connecting cognitive appraisal to final behavioral intention. According to this logic, the positive perception of the meaning of nighttime destination evoked by the cognitive appraisal of authenticity coincides with the willingness and action to undertake critical reflection. Research also confirms that perceiving the meaning of nighttime destination in an activity aligns with subsequent deep cognitive engagement (Kang et al., 2017). Therefore, when university students consider their nighttime destination experience to be of personal importance, they are more likely to engage in thorough examination, questioning, and reconstruction of the cultural information, social interactions, and self-experience it contains. The study proposes the following hypotheses:

**H3.**  *The meaning of nighttime destinations is positively associated with critical reflection.*

**H4.**  *The meaning of nighttime destination mediates the relationship between destination authenticity ([a] objective authenticity, [b] intrapersonal authenticity, and [c] interpersonal authenticity) and the critical reflection.*

Nocturnal escapism is defined as an individual’s tendency to temporarily escape daily pressures and routines through nighttime activities and environments (Semrad & Rivera, 2018). For university students facing heavy academic and social stress, nighttime destinations are often regarded as spaces offering psychological relief. Students with high escapism are more likely to view the nighttime destination experience as a psychological safe zone removed from everyday constraints. In this perceived state of escape, the meaning of nighttime destination they derive from the experience becomes amplified, thereby coinciding with a greater likelihood of lowering psychological defenses and freely engaging in deeper examination and reconstruction of the information, cultural symbols, and self-awareness they encounter. In contrast, students with low escapism may see the destination merely as an ordinary leisure setting, where the meaning of nighttime destination gained from the experience finds it difficult to translate into deep cognitive engagement. Consequently, nocturnal escapism is considered a construct that aligns with the potential of nighttime destinations and strengthens the association between the meaning of nighttime destination and critical reflection. Hence, the following hypothesis is proposed:

**H5.**  *University students’ nocturnal escapism strengthens the positive relationship between the meaning of nighttime destination and their critical reflection.*

**Figure 1:**
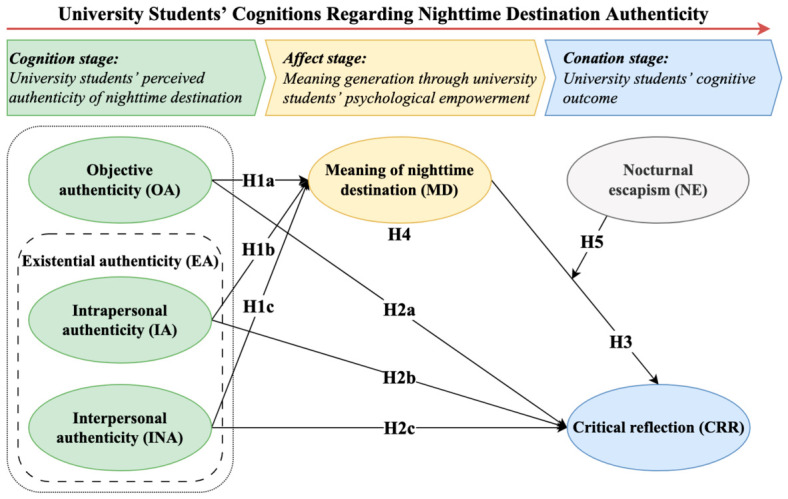
Research model.

On the basis of the hypotheses above, the research model is proposed, as shown in Figure 1.

## 3. Methodology

### 3.1. Nighttime Destination Selection

**Figure 2:**
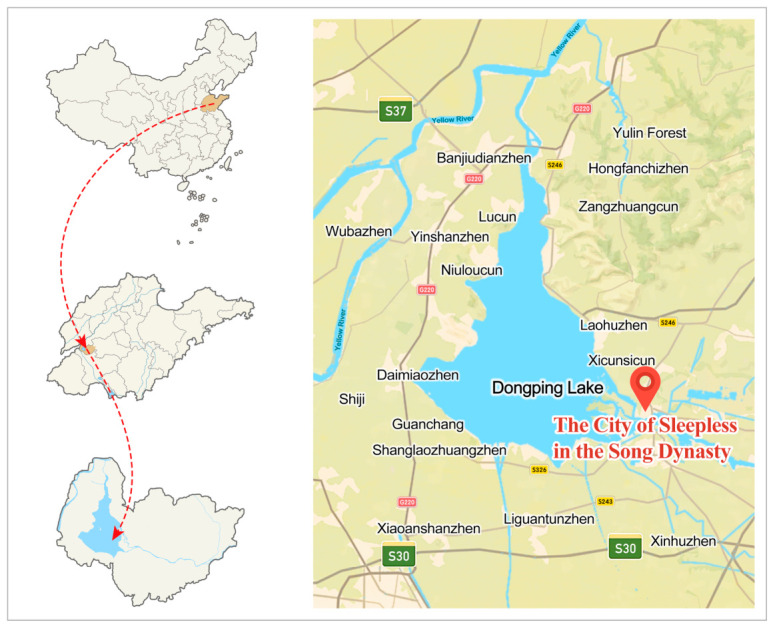
Map indicating the location of ‘The City of Sleepless in the Song Dynasty’.

This study selected the ‘City of Sleepless in the Song Dynasty’ in Taian City, Shandong Province, China (Figure 2) as the research site for two primary reasons, both of which underscore its suitability for investigating cognitive engagement. First, as a large-scale nighttime destination covering approximately 400 acres (about 266,667 m^2^), it constitutes an immersive Song Dynasty-themed district. Its substantial scale and curated environment establish a representative setting, where atmospheric and sensory stimuli are designed to prompt cognitive engagement and critical reflection among university students. Second, it is the only nighttime project in China that integrates the ambiance of an ancient town with a dedicated Song Dynasty-themed cultural district. This distinctive integration creates a unique historical and cultural milieu that provides a rich context for observation. Within this setting, students’ perceptions of both objective and existential authenticity can be naturally and effectively observed, allowing for the examination of how authenticity is cognitively engaged with outside structured classroom environments. Consequently, the destination aligns closely with the study’s objective of investigating the cognitive dynamics through which university students perceive and interpret authenticity within a nighttime destination designed to blend immersive experience with cultural value.

### 3.2. Measurement Development

The questionnaire consisted of three sections (Table A1). It began with two screening questions to confirm that participants were university students aged 18 or above with prior visit experience. The second section contained all latent construct measures. Items were adapted from established scales and assessed using a seven-point Likert scale (1 = strongly disagree, 7 = strongly agree). To address concerns regarding item specificity, this study provides further justification for the measurement approach.

Objective authenticity was measured using items adapted from Kolar and Zabkar (2010). This study prioritized measuring objective authenticity through emotional resonance and environmental immersion (e.g., inspiration, lighting, decorative details) rather than solely focusing on academic historical accuracy. Existential authenticity, comprising intrapersonal and interpersonal dimensions, was adapted from Fu (2019). Each dimension was treated as a first-order construct. The meaning of nighttime destination was measured using items adapted from Kang et al. (2017) to assess the personal significance derived from the experience. Nocturnal escapism was measured using items modified from Semrad and Rivera (2018). This study acknowledges that critical reflection, captured by the scale from Kember et al. (2000), relies on self-report and may overlap with positive post-visit appraisal; however, this instrument is widely validated for distinguishing routine critical reflection. This study recognizes the potential conceptual proximity among authenticity, meaning, escapism, and reflection; however, this study’s discriminant validity checks confirmed that these constructs are empirically distinct enough to warrant separate modeling. Finally, respondents provided demographic data.

To ensure linguistic appropriateness, the questionnaire was translated using a forward-backward translation procedure. Initially, professional translators translated the instrument from English to Chinese. Subsequently, two independent bilingual experts back-translated the Chinese version into English to identify and resolve discrepancies. Following this, two psychology professors evaluated the content validity through a structured expert review. They assessed each item based on its relevance, clarity, and alignment with the construct definitions. Minor wording adjustments were made based on their consensus feedback. A pilot test involving 60 participants was conducted at the ‘City of Sleepless in the Song Dynasty’ in December 2025. The pilot assessed the clarity and comprehensibility of the adapted items, with particular attention to any ambiguity in the authenticity and escapism measures. No issues were identified, suggesting the questionnaire was clear and comprehensive for university students.

### 3.3. Data Collection and Sampling

This study employed purposive sampling to recruit participants visiting the ‘City of Sleepless in the Song Dynasty’. Eligible participants were university students aged 18 or older who had experienced the destination’s nighttime activities. Site management distributed invitation flyers with QR codes to visitors at the main exit plazas. Crucially, the distribution was passive and non-selective: flyers were handed to all exiting visitors regardless of age or student status, and only those who self-identified as eligible university students upon scanning the QR code proceeded to the survey. A total of 800 questionnaires were initially completed via the online platform. After rigorous screening, 32 responses were excluded for completion times under two minutes, and 4 were removed for failing an embedded attention-check item, resulting in 764 valid responses. This yields a valid completion rate of 95.5% (764/800). This high response rate was primarily facilitated by the proactive cooperation of the site management in reaching the target audience, coupled with the convenient, on-site QR code access and the immediate, user-friendly nature of the survey format. No monetary incentives were offered. Data were collected from 20 December 2025 to 2 March 2026. Invalid submissions were rigorously screened: 32 responses were excluded for completion times under two minutes, and 4 were removed for failing an embedded attention-check item (“Please select ‘Strongly Agree’ for this item”), resulting in the final sample of 764. Based on the standard that the sample size should be 5 to 10 times the number of items, according to Hair et al. (2021), and with 22 items in our scale, the target sample size ranged from 110 to 220 participants. The obtained sample of 764 meets this guideline.

This study was conducted in accordance with the ethical principles outlined in the Declaration of Helsinki. Although formal ethical review was not required for this type of non-invasive, anonymous survey under national regulations, this study ensured rigorous adherence to ethical standards. Informed consent was obtained electronically from all participants prior to their commencement of the survey, which explicitly stated the study’s purpose, assured anonymity, and emphasized that participation was entirely voluntary. Data protection was strictly maintained: all responses were anonymized upon submission, stored on a secure server with restricted access, and no identifying information was linked to the dataset. Regarding the involvement of site management in distributing flyers, this study acknowledges that this institutional support could raise concerns regarding sampling independence or response bias. However, this study took specific measures to mitigate this risk. The site management acted solely as a facilitator for physical distribution and had no access to the survey content, the data collection platform, or the respondents’ identities. Participation was entirely voluntary, with no pressure or incentive provided by the management. Furthermore, the anonymous nature of the survey and the immediate electronic submission upon exit ensured that the management could not track or influence individual responses. Therefore, this study is confident that the data collection remained independent and that the responses accurately reflect the participants’ genuine experiences.

Table A2 outlines the demographic profile of respondents from the ‘City of Sleepless in the Song Dynasty’. The sample is predominantly young (87.18% aged 18–24) and evenly split in gender (59.95% female, 40.05% male), with half (50.00%) being bachelor’s students engaged in formal higher education. This composition is pertinent for examining critical reflection. Most notably, the distribution of academic majors is heavily weighted toward fields concerned with cultural interpretation: 52.75% specialize in humanities and social sciences, and 26.31% in art and design. This strong representation ensures the sample is particularly attuned to the historical and atmospheric cues of the Song Dynasty-themed destination. Consequently, it is well suited for investigating how authenticity is perceived in a designed nighttime destination, although this concentration also constitutes a potential source of selection bias. The over-representation of students from culture, aesthetics, and social contexts may limit the generalizability of findings to those in science, technology, engineering, and mathematics or other less culturally oriented disciplines. Consequently, the findings regarding cognitive engagement arising from situated experience should be interpreted within this specific disciplinary context.

### 3.4. Data Analysis

This two-phase quantitative study adopts a mixed-method analytical approach, combining Partial Least Squares Structural Equation Modeling (PLS-SEM) and fuzzy-set Qualitative Comparative Analysis (fsQCA 3.0) to examine how perceptions of authenticity in a nighttime destination associate with university students’ cognition, particularly their critical reflection. The first phase utilized PLS-SEM to test the research hypotheses, a method chosen for its ability to handle the study’s complex and predictive model without imposing restrictive distributional assumptions (Chin, 1998; Hair, 2014). This makes it particularly suitable for exploratory research in domains like university students’ cognitions, where theoretical foundations are still developing (Hair et al., 2017). Furthermore, PLS-SEM offers high predictive power, aligning with the study’s aim to test theoretical assumptions and assess cultural impact (Shmueli et al., 2019). Using SmartPLS 4.1.1.1, bootstrapping with 5000 subsamples was performed to generate standard errors and confidence intervals for the path coefficients, allowing for the examination of statistical significance without relying on parametric assumptions (Hair et al., 2012). This analytical procedure enabled a rigorous test of the study’s direct and mediating effects.

While PLS-SEM is effective for examining average net associations between constructs, its linear and symmetric nature may not fully capture configurational complexity, such as equifinality or asymmetric associations, wherein multiple combinations of conditions can be consistently associated with the same outcome (Bollen & Pearl, 2013; Huang et al., 2026; Woodside, 2013). To address these limitations, the second phase applied fsQCA, which analyzes how combinations of conditions jointly influence an outcome. This multiple approach provides a more complete understanding: PLS-SEM identifies general predictive effects, whereas fsQCA reveals the specific configurations of conditions consistently associated with high critical reflection.

## 4. Phase One: Model Testing Results

### 4.1. Measurement Model Evaluation

The measurement model had acceptable psychometric characteristics. Table 1 indicates that all factor loadings were above 0.7 (Hair et al., 2019). Convergent validity was confirmed, as the average variance extracted (AVE) for each construct ranged from 0.727 to 0.775, surpassing the 0.50 threshold (Hair et al., 2021). Additionally, both Cronbach’s α and composite reliability (CR) values ranged from 0.817 to 0.883, all exceeding the recommended minimum of 0.7 (see Table 2). Discriminant validity was established by two criteria: the square root of each construct’s AVE exceeded its correlations with other constructs, and all heterotrait–monotrait (HTMT) ratios were below 0.90 (Henseler et al., 2015). Subsequent analyses revealed that common method bias was not a substantial issue, as Harman’s single-factor test explained merely 39.644% of the variation, well below the 50% threshold (Fuller et al., 2016). Furthermore, all variance inflation factor (VIF) values were beneath 5, signifying the absence of significant multicollinearity. However, consistent with the limitations noted by Podsakoff et al. (2003), we acknowledge that statistical tests cannot fully exclude the possibility of CMB. While the results suggest that CMB is unlikely to pose a severe threat to the validity of our findings, readers should interpret the results with this caveat in mind.

### 4.2. Structural Model Assessment and Hypothesis Verification

**Figure 3:**
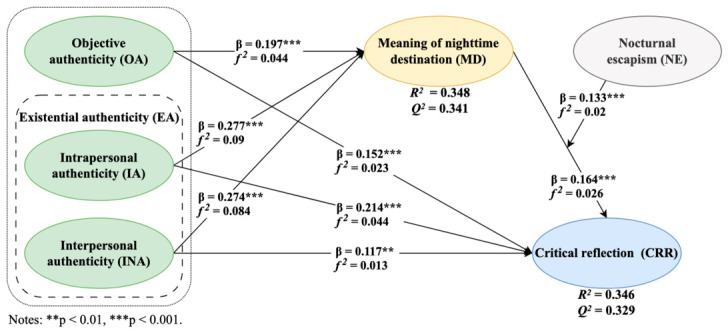
SEM results.

Figure 3 and Table 3 present the direct path estimations for the hypothesized model. The results indicate that perceived objective authenticity (β = 0.197, *p* < 0.001; f^2^ = 0.044), intrapersonal authenticity (β = 0.277, *p* < 0.001; f^2^ = 0.090), and interpersonal authenticity (β = 0.274, *p* < 0.001; f^2^ = 0.084) are each significantly and positively associated with the meaning of nighttime destination. The effect sizes (f^2^ ≥ 0.02) suggest that these associations are non-negligible, thereby fully corroborating H1a, H1b, and H1c. Regarding H2, the analysis reveals that objective authenticity (β = 0.152, *p* < 0.001; f^2^ = 0.023) and intrapersonal authenticity (β = 0.214, *p* < 0.001; f^2^ = 0.044) are significantly associated with critical reflection, aligning with H2a and H2b. The path linking interpersonal authenticity to critical reflection was statistically significant (β = 0.117, *p* < 0.01) but associated with a negligible effect size (f^2^ = 0.013 < 0.02; Hair et al., 2019). Thus, while the statistical association exists, it lacks substantive magnitude, and H2c is not substantively supported. Finally, supporting H3, the meaning of nighttime destinations among university students is significantly associated with critical reflection (β = 0.164, *p* < 0.001; f^2^ = 0.026).

Figure 3 and Table 3 demonstrate that the R^2^ values for the meaning of nighttime destination (0.348) and critical reflection (0.346) surpass the suggested threshold of 0.10, signifying substantial explanatory power (Cohen, 2013). The Stone–Geisser Q^2^ values for the meaning of nighttime destination (0.341) and critical reflection (0.329) were both substantially greater than zero (Q^2^ > 0), confirming the model’s predictive relevance for these endogenous constructs (Geisser, 1975; Stone, 1974). Specifically, these values exceeded the 0.15 threshold, indicating moderate predictive relevance according to conventional PLS-SEM benchmarks (Hair et al., 2019). The standardized root mean square residual (SRMR) of the model is 0.04 (<0.08), signifying an adequate model fit.

To test the mediation effects, 5000 bootstrapped samples with bias-corrected 95% confidence intervals (CI) were employed, following established procedures for complex path modeling (Hayes, 2022). As shown in Table 4, the indirect effects of objective authenticity (β = 0.032, 95% CI [0.014, 0.054]), intrapersonal authenticity (β = 0.045, 95% CI [0.021, 0.073]) and interpersonal authenticity (β = 0.045, 95% CI [0.022, 0.071]) on critical reflection, mediated by the meaning of nighttime destination, were statistically significant as their confidence intervals did not include zero (Cohen et al., 2013). Therefore, H4 was supported. A further examination of the mediation types, informed by the direct effects, reveals a nuanced picture. For objective and intrapersonal authenticity, the significant indirect effects coexist with their significant direct effects on critical reflection (with f^2^ ≥ 0.02), indicating partial mediation. In contrast, for interpersonal authenticity, while its indirect effect is significant, its hypothesized direct effect on critical reflection was rejected due to a negligible effect size (f^2^ = 0.013 < 0.02) despite a significant *p*-value. In contrast, for interpersonal authenticity, while the indirect effect via the meaning of nighttime destination is significant, the hypothesized direct effect on critical reflection requires a more nuanced interpretation. Although the direct path remains statistically significant (*p* < 0.01), its effect size is negligible (f^2^ = 0.013 < 0.02; Hair et al., 2019). Consequently, the relationship between interpersonal authenticity and critical reflection is primarily, though not exclusively, transmitted through the meaning of nighttime destination. While the data support a substantial mediation effect, the persistence of a statistically significant yet substantively weak direct path suggests that the full mediation conclusion should be tempered.

**Figure 4:**
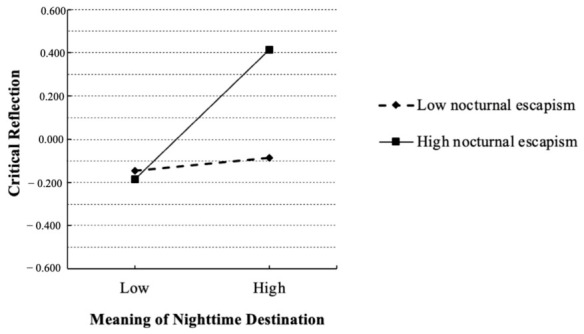
Simple slope analysis.

Figure 3 shows that the moderating effect of nocturnal escapism on the relationship between the meaning of nighttime destination and critical reflection was significant (β = 0.133, *p* < 0.001; f^2^ = 0.02 ≥ 0.02). Therefore, H5 was supported. The findings from the simple slope analysis (see Figure 4) elucidate the nature of the moderating effect. Specifically, for university students with high nocturnal escapism, there is a strong positive relationship between the meaning of nighttime destination and their critical reflection. In contrast, for those with low nocturnal escapism, the level of critical reflection remains consistently low and shows minimal increase even as the perceived meaning of the destination rises, resulting in a relatively flat slope. This pattern indicates that nocturnal escapism acts as a crucial boundary condition. The positive cognitive benefit of finding meaning of nighttime destination (i.e., enhanced critical reflection) is significantly amplified for individuals with high levels of nocturnal escapism. Conversely, for university students with low nocturnal escapism, the meaning of nighttime destination has a markedly weaker—almost negligible—impact on fostering critical reflection.

## 5. Phase Two: FsQCA Results

### 5.1. Data Calibration

Prior to performing the fsQCA, data calibration was carried out in accordance with established protocols (Ragin, 2009). Raw data were converted into fuzzy-set membership scores ranging from 0.0 (full non-membership) to 1.0 (full membership), with 0.5 representing the threshold of maximal ambiguity (Schneider & Wagemann, 2012). Following methodological conventions (Fiss, 2011; Greckhamer et al., 2018), this study employed the 75th percentile as the anchor for full membership, the mean as the crossover point (0.5), and the 25th percentile as the anchor for full non-membership. This choice is justified because the scales used in this study are continuous and normally distributed, making the mean and quartiles theoretically meaningful representations of set boundaries (Greckhamer et al., 2018). Calibration was performed using fsQCA 3.0 software.

### 5.2. Necessary Analysis

A necessary analysis was conducted following the calibration of the data into fuzzy sets. Based on the theoretical framework and structural model results, this study posited that all five antecedent conditions (OA, IA, INA, MD, NE) would positively contribute to high critical reflection. The outcome condition was delineated as high critical reflection, and each antecedent condition was evaluated for necessity. Consistency measures the extent to which cases correspond with a requisite relationship, ranging from 0 to 1 (Rihoux, 2006). According to established convention, a condition is deemed necessary if its consistency is above 0.90 (Ragin, 2009). As shown in Table 5, all five antecedent conditions displayed consistency scores ranging from 0.345 to 0.763, each falling below the 0.90 threshold. These results indicate that no individual condition is necessary for high critical reflection. Consequently, the conditions advanced to further configurational analysis to assess their combined impact on the outcome.

### 5.3. Truth Table Construction

Following Ragin (2009), the five antecedent conditions theoretically yield 32 (2^5^) logically possible configurations. Configurations failing to meet the frequency threshold of five cases and the PRI consistency benchmark of 0.75 were excluded from subsequent analysis. While all 32 rows satisfied the frequency threshold, only 5 rows met the PRI consistency cutoff of 0.75. Notably, the fifth row exhibited a raw consistency of 0.875, a PRI consistency of 0.790, and a SYM consistency of 0.797. Among the four solutions generated by fsQCA, the intermediate solution was selected for interpretation. This choice is justified because it maintains the optimal balance between analytical clarity and explanatory completeness (Fiss, 2011). As reported in Table 6, four distinct configurations lead to high critical reflection. These four configurations jointly provide a high level of explanatory power and consistency, as indicated by the overall solution coverage, which is 0.585, and the overall solution consistency, which is 0.887. The first two solutions, S1a and S1b, both have consistency ratings of 0.938 and 0.934, and raw coverage scores of 0.507 and 0.485, respectively. These results indicate that the core conditions of OA, IA, and INA are shared by both of these solutions. The peripheral conditions of S1a and S1b are distinct from one another: MD for S1a and NE for S1b. S2a and S2b are both configured with IA, MD, and NE as core conditions. The consistency scores for S2a and S2b are 0.920 and 0.928, while the raw coverage scores for S2a and S2b are 0.496 and 0.489, respectively. The difference between them rests in the peripheral condition, which is identified as OA for S2a and INA for S2b.

To ensure the reliability of the findings, this study conducted robustness checks by adjusting the PRI consistency threshold. Although the primary analysis utilized a stringent PRI cutoff of 0.75, we re-estimated the models using a slightly relaxed threshold of 0.70. Specifically, the combinations of core conditions (e.g., the presence of OA, IA, and INA in S1a/S1b, and IA, MD, NE in S2a/S2b) persisted across both thresholds. While minor variations in peripheral conditions were observed, the overall solution consistency and coverage remained within acceptable ranges (consistency > 0.85, coverage > 0.55). These results confirm that the identified pathways to high critical reflection are robust and not merely artifacts of a specific PRI cutoff.

## 6. General Discussion

Drawing on the integrated findings from Partial Least Squares Structural Equation Modeling (PLS-SEM) and fuzzy-set Qualitative Comparative Analysis (fsQCA), this study explores the cognitive patterns of university students within nighttime destination. The PLS-SEM results corroborated the hypothesized pathways grounded in the cognition–affect–conation framework, revealing the average net associations among the constructs. Complementarily, the fsQCA identified four distinct configurations of antecedent conditions consistently associated with high levels of critical reflection, highlighting the configurational complexity and the presence of equifinality.

Regarding the direct paths, the results indicate that perceptions of objective, intrapersonal, and interpersonal authenticity are each significantly and positively associated with the meaning of nighttime destination. This aligns with the views of Kolar and Zabkar (2010) and Fu (2019), suggesting that different dimensions of authenticity coincide with complementary pathways for individuals to construct personal meaning within nighttime destination. Concerning the associations with critical reflection, both objective and intrapersonal authenticity demonstrated significant and substantive positive associations. However, the path from interpersonal authenticity to critical reflection, while statistically significant, yielded a negligible effect size and thus lacks substantive support. A plausible interpretation is that, within the specific context of a nighttime destination, the association between social interaction and critical reflection appears to operate primarily through the construct of personal meaning. In the absence of such internalization, social interaction does not necessarily coincide with profound critical reflection.

The mediation analysis shows that the meaning of nighttime destination accounts for part of the covariance between all three authenticity perceptions and critical reflection, albeit with different patterns. For objective and intrapersonal authenticity, meaning accounts for a portion of the total association. This indicates that beyond coinciding with critical reflection via enhanced meaning, these two authenticity dimensions also share direct associations independent of meaning. This finding resonates with the internal logic of psychological empowerment theory (Llorente-Alonso et al., 2024) and the cognition–affect–conation framework (Bagozzi, 1992). In contrast, for interpersonal authenticity, the association is primarily transmitted through meaning. The results indicate substantial mediation, implying that the link between interpersonal interaction and critical reflection is largely dependent on its association with personalized meaning construction. This predominantly indirect effect strongly supports the argument of Kang et al. (2017) that the perception of meaning in an activity coincides with subsequent deep cognitive engagement.

The test of the moderating effect confirms that nocturnal escapism moderates the strength of the association between destination meaning and critical reflection. Simple slope analysis further illustrates that for students with high escapism tendencies, the association between meaning and critical reflection is stronger, whereas for those with low escapism, this association is minimal. This result clarifies that nocturnal escapism acts as a crucial boundary condition. It coincides with the framing of the nighttime destination as a psychological space where students may temporarily shed daily pressures and psychological defenses (Semrad & Rivera, 2018). In this perceived state, the personal meaning derived from the experience is significantly amplified, coinciding with a smoother transformation into deep examination and reconstruction of information, cultural symbols, and self-awareness.

Finally, the configurational findings from fsQCA provide a complementary perspective to the average-effect-focused PLS-SEM results, revealing multiple equifinal paths consistently associated with high critical reflection. The analysis identifies four core condition configurations (S1a, S1b, S2a, S2b), with satisfactory overall solution consistency and coverage. First, all four paths include intrapersonal authenticity as a core condition. This, from a configurational viewpoint, reinforces the PLS-SEM finding, underscoring the robust association between self-exploration experiences and deep cognition. Second, the results clearly demonstrate the characteristics of multiple conjunctural causation and equifinality. For instance, S1a and S1b show that when perceptions of objective, intrapersonal, and interpersonal authenticity are all present, high critical reflection is observed whether accompanied by high meaning (S1a) or high nocturnal escapism (S1b). S2a and S2b indicate that based on the core combination of intrapersonal authenticity, meaning, and nocturnal escapism, objective authenticity (S2a) and interpersonal authenticity (S2b) can substitute for each other as peripheral conditions. These configurations align with the perspective of complexity theory (Woodside, 2013), suggesting that the emergence of high-order cognitive outcomes coincides with the complex interplay and substitutability among antecedent conditions, rather than isolated linear associations.

## 7. Implications, Limitations and Future Research Directions

### 7.1. Theoretical Implications

This study’s theoretical contributions primarily center on two areas. First, this study empirically tests and enriches the explanatory power of psychological empowerment theory regarding motivational dynamics. The results suggest that the perceived meaning of nighttime destination acts as a central correlate of an individual’s psychological empowerment. It plays a crucial mediating role between authenticity perceptions and critical reflection. Particularly, the research reveals distinct patterns through which different authenticity dimensions exert their influence via meaning: for interpersonal authenticity, its effect is transmitted through this psychological empowerment state, whereas for objective and intrapersonal authenticity, meaning serves as a partial mediator. This finding clarifies the pathways of psychological empowerment, indicating that under certain conditions, empowerment is a necessary conduit for stimulating deep cognitive engagement, while under other conditions, environmental stimuli may also directly initiate cognitive investment. This provides a more nuanced lens for understanding the conditional and complex nature of the empowerment state (Llorente-Alonso et al., 2024).

Second, this study verifies and refines the logical sequence of the cognition–affect–conation (CAC) framework in explaining high-order cognitive outcomes (Bagozzi, 1992). By framing authenticity perceptions as a cognitive appraisal, the meaning of nighttime destination as an affective response, and critical reflection as a conative outcome, this study successfully constructs and validates a complete CAC pathway model. This not only provides a clear theoretical outline of the sequential psychological state from environmental cognition to deep reflection but, more importantly, the mediation findings offer strong evidence for the indispensable bridging role of affect in this sequence. Furthermore, the discovery of the moderating effect of nocturnal escapism introduces a significant boundary condition to this framework. It shows that the strength of the transformation from affect to conation is influenced by individual psychological motivational traits, thereby rendering the framework’s application more contextualized and multidimensional.

In summary, the theoretical contribution of this research lies in constructing a comprehensive framework for understanding cognitions in nighttime destinations through cross-theoretical integration and empirical testing. It refines the operational mechanisms of psychological empowerment theory and validates and enriches the application scenarios of the CAC framework. More importantly, it demonstrates that organically combining these theoretical perspectives enables a more comprehensive and in-depth revelation of the multiple antecedents and interactive influences behind cognitive phenomena in complex environments.

### 7.2. Practical Implications

This study proposes tentative practical insights for two key groups: designers and managers of nighttime projects, and university students themselves.

On the one hand, there are implications for nighttime tourism destination planners and managers. The results suggest that destination authenticity may serve as a catalyst for stimulating deep cognitive engagement. Thus, managers may consider balancing both objective and existential authenticity in project design and narrative creation. Specifically, in environmental design (e.g., architecture, lighting, performances), emphasis could be placed on historical and cultural accuracy to provide credible details, which can offer material for critical questioning and comparison, especially for young students. More crucially, it is essential to design elements that facilitate existential authentic experiences. For instance, rather than generic interactive activities, managers might install specific, introspection-oriented features such as AR-enabled historical decoding stations or “Reflection Corners” equipped with sensory deprivation pods and digital journaling walls to encourage solitary contemplation and self-dialog. Organizing thematic activities or social scenarios requiring teamwork and deep interaction may foster interpersonal authenticity. To translate this into personal meaning, managers could implement structured programs like “Perspective Exchange Salons” or “Cultural Debate Circles” where visitors might collaboratively analyze historical events or design solutions to contemporary issues based on the heritage context, moving beyond simple collective entertainment.

On the other hand, there are implications for the university student population. The conclusions may offer a reference for how students might derive personal growth from leisure tourism. Students could recognize that a positive, deep experience is not passive reception but an active act of construction. When visiting nighttime destinations, they might consciously observe environmental details, engage themselves in feeling and exploring, and engage in genuine communication with peers, while contemplating the personal significance of the place or experience. Experiencing this meaning of nighttime destination presents an opportune moment to initiate critical reflection. Understanding the positive role of nocturnal escapism, students may also view their need to relieve stress through nighttime activities more positively. They could attempt to transform this relaxed and open psychological state into a valuable opportunity for independent thinking about what they encounter, thereby gaining cognitive growth alongside entertainment.

### 7.3. Limitations and Future Research Directions

This study acknowledges several limitations that outline specific pathways for future inquiry. First, the reliance on a non-probability, convenience sampling strategy at a single cultural heritage site limits the generalizability of the findings. The over-representation of students from humanities and arts disciplines, coupled with the specific socio-cultural context of the ‘City of Sleepless in the Song Dynasty’, means the results may not extend to broader student populations. Additionally, data were collected exclusively during the winter season (December to March), which may introduce seasonal bias, as colder weather and holiday periods could influence visitation moods, perceptions of atmosphere, and engagement levels. Future research should employ multi-site sampling across varied cultural contexts to disentangle universal cognitive dynamics. Specifically, replication studies in non-Chinese and non-heritage nighttime contexts (e.g., modern urban entertainment districts or natural nightscapes) are needed to test the cross-cultural robustness and contextual boundaries of the proposed model.

Second, the cross-sectional design and the inherent limitations of self-reported data constrain the interpretability of the findings. While this study draws upon learning theories, the inability to infer order prevents us from establishing whether perceived authenticity temporally precedes meaning-making, or vice versa. Moreover, the exclusive use of self-report measures for critical reflection raises concerns regarding common method variance and social desirability bias. Although statistical remedies were applied, self-reports may not fully capture the non-conscious or procedural dimensions of cognitions. Future studies should move beyond the sole moderator of nocturnal escapism by incorporating stable dispositional variables, such as openness to experience or the need for cognition, to examine how individual differences moderate the pathway from authenticity to reflection. Furthermore, longitudinal designs or experience-sampling methodologies are recommended to trace the long-term effects of nighttime destination visits on university students’ sustained reflective practice, moving beyond the static snapshot captured in this study. Integrating multi-modal data—such as eye-tracking, think-aloud protocols, or psychophysiological measures—would also yield a more nuanced account of how experiential authenticity fuels critical intellectual development.

Third, several measurement and procedural limitations warrant consideration. Although the site management’s involvement was limited to physical flyer distribution, the potential for demand characteristics cannot be entirely dismissed, as participants may have subconsciously aligned their responses with perceived institutional expectations. Additionally, while the nocturnal escapism scale (Semrad & Rivera, 2018) provided a robust measure, it was originally developed in a festival tourism context. Its application in a nighttime cultural heritage destination represents a contextual extension, and thus future research is needed to further substantiate its applicability and stability within such settings. Finally, the sample’s disciplinary skew reinforces the need for caution when generalizing these findings to students in science, technology, engineering, and mathematics or other less culturally oriented disciplines.

## Figures and Tables

**Table 1:** Basic information of constructs.

Construct/Items		Mean	S.D.	Factor Loadings	VIF
Objective authenticity (OA)					
OA1		4.723	1.571	0.858	2.21
OA2		4.732	1.579	0.865	2.251
OA3		4.747	1.564	0.853	2.153
OA4		4.738	1.598	0.859	2.351
Existential authenticity (EA)	Intrapersonal authenticity (IA)				
	IA1	4.726	1.601	0.856	2.169
	IA2	4.666	1.589	0.845	2.067
	IA3	4.681	1.598	0.854	2.224
	IA4	4.703	1.565	0.861	2.212
	Interpersonal authenticity (INA)				
	INA1	4.836	1.502	0.861	1.803
	INA2	4.899	1.482	0.85	1.805
	INA3	4.826	1.572	0.856	1.818
Meaning of nighttime destination (MD)					
MD1		4.767	1.608	0.883	2.131
MD2		4.733	1.647	0.886	2.124
MD3		4.804	1.605	0.872	2.115
Nocturnal escapism (NE)					
NE1		4.914	1.596	0.854	2.135
NE2		4.89	1.581	0.842	2.152
NE3		4.835	1.547	0.847	2.113
NE4		4.946	1.579	0.868	2.221
Critical reflection (CRR)					
CRR1		4.755	1.591	0.852	2.182
CRR2		4.758	1.588	0.856	2.172
CRR3		4.738	1.615	0.854	2.195
CRR4		4.711	1.624	0.856	2.146

**Table 2:** Measurement model test results.

Construct	Cronbach’s Alpha	CR	AVE	Fornell-Larcker Criterion/HTMT					
				OA	IA	INA	MD	NE	CRR
OA	0.881	0.883	0.737	**0.859**	0.470	0.532	0.499	0.512	0.486
IA	0.876	0.877	0.729	0.413	**0.854**	0.493	0.546	0.566	0.537
INA	0.817	0.818	0.732	0.452	0.417	**0.855**	0.571	0.563	0.489
MD	0.855	0.858	0.775	0.435	0.473	0.479	**0.881**	0.511	0.492
NE	0.875	0.879	0.727	0.45	0.495	0.478	0.444	**0.853**	0.449
CRR	0.877	0.877	0.730	0.429	0.472	0.414	0.428	0.395	**0.855**

Note: (1) Fornell and Larcker: the diagonal elements (in bold) represent the square root of the AVE, while the off-diagonal elements below the diagonal are the inter-construct correlations. (2) Heterotrait–monotrait ratio (HTMT) values are displayed above the diagonal.

**Table 3:** Path analysis results.

Hypothesis/Path	β	Standard Error	t	*p*	f^2^	95% Confidence Interval		Results
						LL	UL	
H1a. OA → MD	0.197	0.038	5.228	<0.001 ***	0.044	0.12	0.269	Supported
H1b. IA → MD	0.277	0.036	7.726	<0.001 ***	0.09	0.205	0.347	Supported
H1c. INA → MD	0.274	0.035	7.788	<0.001 ***	0.084	0.204	0.343	Supported
H2a. OA → CRR	0.152	0.04	3.816	<0.001 ***	0.023	0.071	0.228	Supported
H2b. IA → CRR	0.214	0.041	5.289	<0.001 ***	0.044	0.134	0.294	Supported
H2c. INA → CRR	0.117	0.042	2.798	0.005 **	0.013	0.032	0.198	Rejected
H3. MD → CRR	0.164	0.041	3.996	<0.001 ***	0.026	0.083	0.243	Supported
MD	R^2^: 0.348; Q^2^: 0.341							
CRR	R^2^: 0.346; Q^2^: 0.329							

Notes: ** *p* < 0.01, *** *p* < 0.001.

**Table 4:** Mediation analysis results.

Path (H4)	β	Standard Error	t	*p*	95% Confidence Interval		Results
					LL	UL	
OA → MD → CRR	0.032	0.01	3.167	0.002 **	0.014	0.054	Partial mediation
IA → MD → CRR	0.045	0.013	3.455	0.001 **	0.021	0.073	Partial mediation
INA → MD → CRR	0.045	0.012	3.604	<0.001 ***	0.022	0.071	Substantial mediation

Notes: ** *p* < 0.01, *** *p* < 0.001.

**Table 5:** Analysis of necessary conditions for predicting critical reflection.

Conditions	High Critical Reflection		Low Critical Reflection	
	Consistency	Coverage	Consistency	Coverage
OA	0.725	0.730	0.393	0.377
~OA	0.381	0.397	0.718	0.713
IA	0.736	0.725	0.405	0.380
~IA	0.370	0.396	0.706	0.718
INA	0.700	0.756	0.345	0.354
~INA	0.402	0.392	0.763	0.707
MD	0.727	0.719	0.396	0.373
~MD	0.367	0.390	0.702	0.709
NE	0.687	0.733	0.368	0.374
~NE	0.412	0.407	0.737	0.691

**Table 6:** Main configurations for high critical reflection.

Configuration	Solutions			
	S1a	S1b	S2a	S2b
Objective authenticity (OA)	●	●	●	
Intrapersonal authenticity (IA)	●	●	●	●
Interpersonal authenticity (INA)	●	●		●
Meaning of nighttime destination (MD)	●		●	●
Nocturnal escapism (NE)		●	●	●
Raw coverage	0.507	0.485	0.496	0.489
Unique coverage	0.043	0.021	0.032	0.025
Consistency	0.938	0.934	0.920	0.928
Solution coverage	0.585			
Solution consistency	0.887			

Note: ● represents the presence of peripheral conditions, ● represents the presence of core conditions, and a blank space indicates a “don’t care” condition.

## Data Availability

The raw data supporting the conclusions of this article will be made available by the authors on request. Public sharing is limited in accordance with the data management and sovereignty policies of the authors’ institution, which govern the stewardship of research outputs. While the survey was anonymous and collected no personal identifiers, the complete dataset is maintained under institutional guidelines. We are committed to facilitating transparent research and will provide the data to qualified researchers for verification purposes under a standard data access agreement that ensures appropriate use and citation.

## Appendix A

**Table A1:** Measurement items.

Constructs		Items	Description	Sources
Objective authenticity (OA)		OA1	The overall nighttime atmosphere and lighting design of the destination inspired me.	Kolar and Zabkar (2010)
		OA2	I liked the distinctive lighting and decorative details within the nighttime setting.	
		OA3	I liked the way the site blends with the surrounding nighttime landscape/historical area/townscape, which offers many other interesting sights after dark.	
		OA4	I found the information and stories presented through the nighttime experience engaging and interesting.	
Existential authenticity (EA)	Intrapersonal authenticity (IA)	IA1	In the nighttime atmosphere, I felt freed from the routines and constraints of daily life and became more connected to my own feelings and desires.	Fu (2019)
		IA2	During the nighttime visit, I gave myself the chance to freely explore and understand what interested me, such as local culture, activities, or surroundings.	
		IA3	Throughout the nighttime experience, I did not simply follow others’ expectations but engaged in a way that felt true to my own style and preferences.	
		IA4	I sought out extraordinary or unusual experiences in the nighttime setting to fulfill a sense of personal satisfaction or self-realization.	
	Interpersonal authenticity (INA)	INA1	During the nighttime visit, I interacted with local residents in a natural, authentic, and friendly manner.	Fu (2019)
		INA2	During the nighttime visit, I connected with my family members in a relaxed and genuine way.	
		INA3	During the nighttime visit, I engaged with other visitors around me in a friendly and unaffected way.	
Meaning of nighttime destination (MD)		MD1	The nighttime destination is very important to me.	Kang et al. (2017)
		MD2	The nighttime destination is meaningful to me.	
		MD3	The nighttime destination activities are personally meaningful to me.	
Nocturnal escapism (NE)		NE1	Being in this nighttime environment makes me feel like I am in another world.	Semrad and Rivera (2018)
		NE2	Engaging in nocturnal activities here helps me get away from it all.	
		NE3	I become so emotionally involved in the nighttime experience that I forget everything else.	
		NE4	I find myself completely immersed in the nighttime environment here.	
Critical Reflection (CRR)		CRR1	After this nighttime experience, I have reconsidered how I see myself.	Kember et al. (2000)
		CRR2	This visit has challenged some of my previously held perspectives.	
		CRR3	Following this experience, I have reflected on changing my usual way of doing things.	
		CRR4	During this visit, I recognized limitations in what I had once believed to be right.	

**Table A2:** Sample profile.

Demographic	Item	Frequency (%)
Gender	Male	306 (40.05)
	Female	458 (59.95)
Age	18–21	454 (59.43)
	22–24	212 (27.75)
	25–30	95 (12.43)
	Above 30	3 (0.39)
Education levels	Junior college	157 (20.55)
	Bachelor’s degree	382 (50.00)
	Master’s degree	211 (27.62)
	Doctoral degree	14 (1.83)
University major/academic field	Humanities and social sciences	403 (52.75)
	Science and engineering	19 (2.49)
	Business and management	113 (14.79)
	Art and design	201 (26.31)
	Others (i.e., medicine, agriculture, physical education)	28 (3.66)
